# Supplementary figures and images for: Survival and Enrichment Analysis of Epithelial–Mesenchymal Transition Genes in Bladder Urothelial Carcinoma
Source: Genes (Basel). 2023 Sep 30;14(10):1899. doi: 10.3390/genes14101899 (PMC10606556; doi:10.3390/genes14101899)

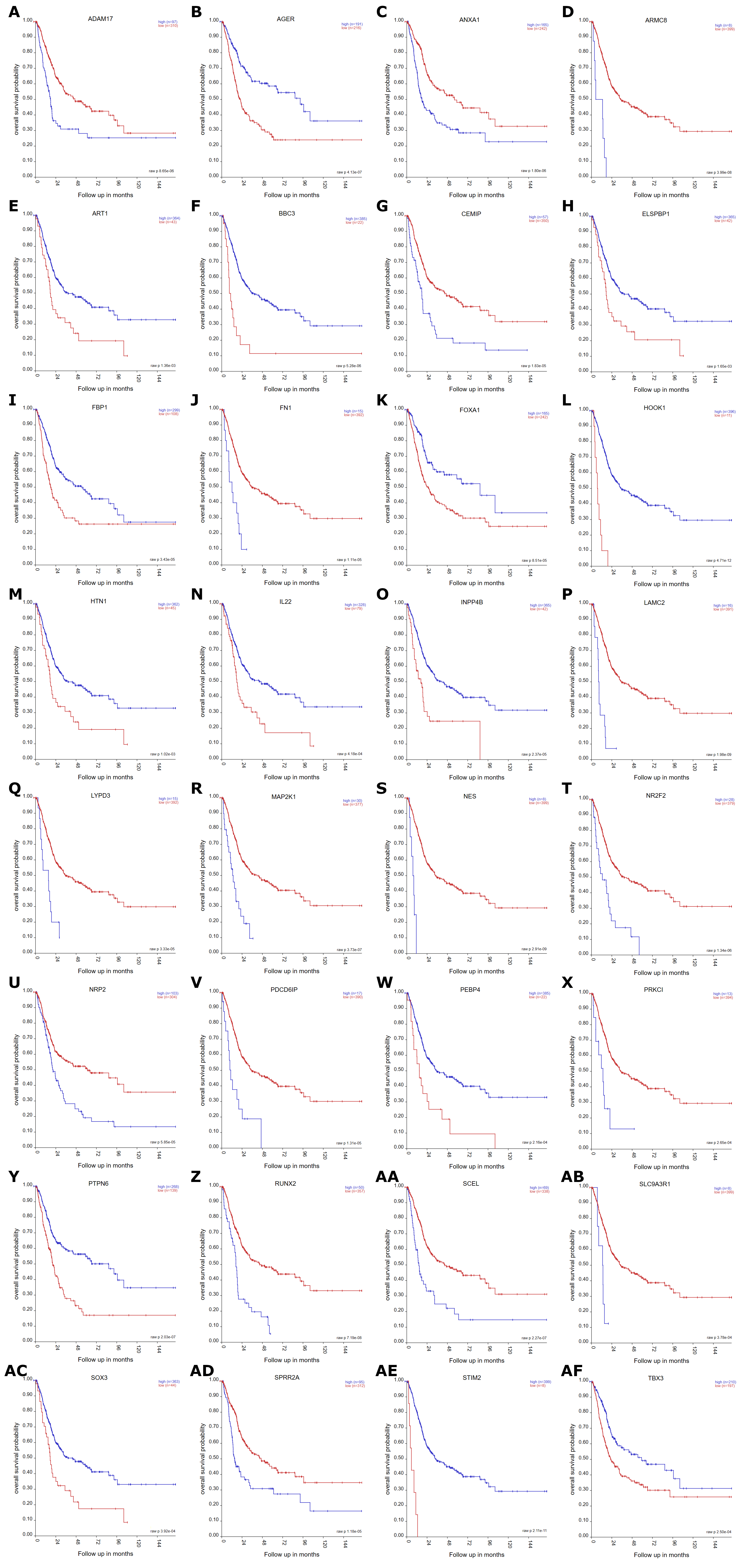

Supplement: Supplementary file 1 [file genes-14-01899-s001.zip › Supplemental Figure S1.png]

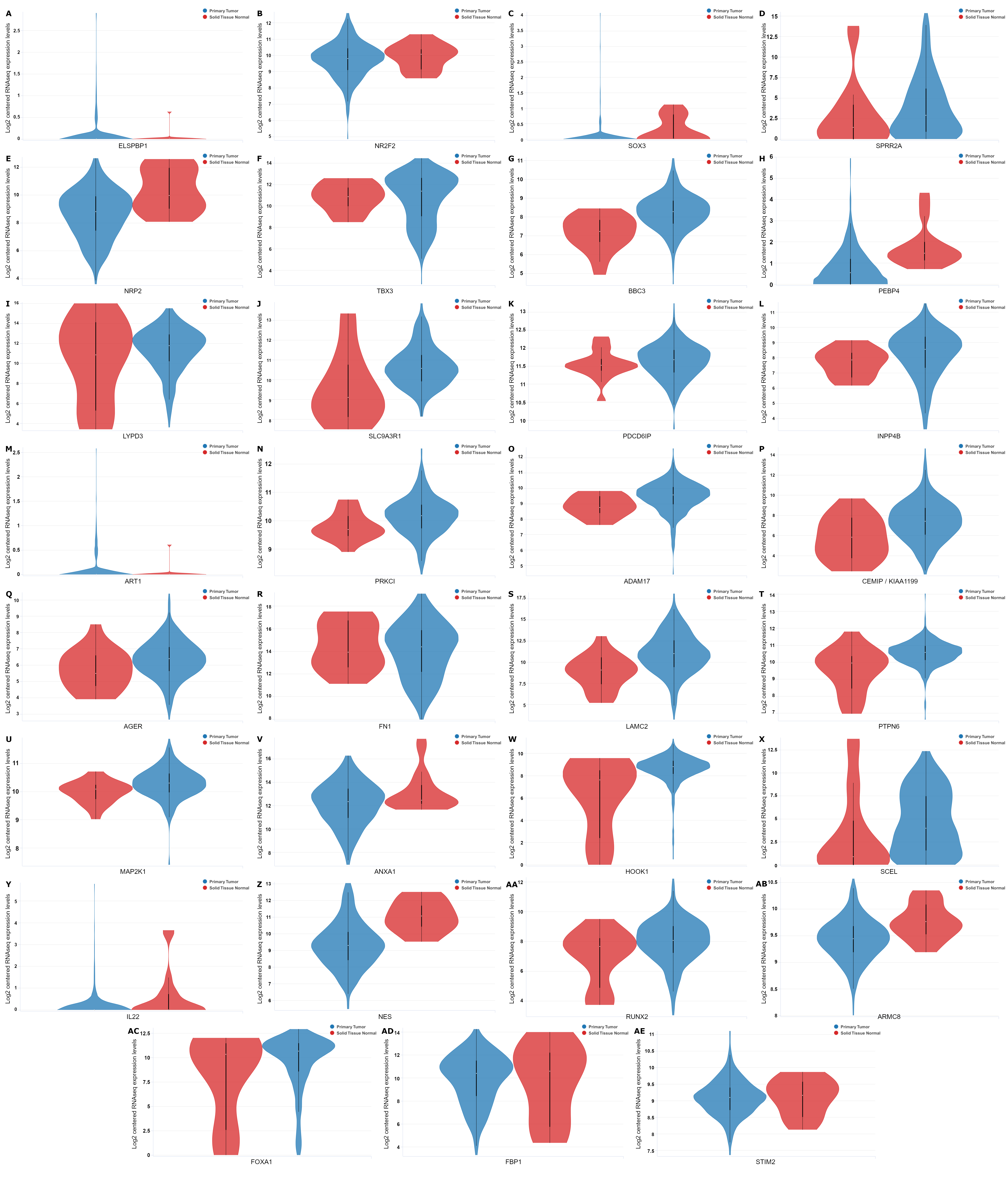

Supplement: Supplementary file 1 [file genes-14-01899-s001.zip › Supplemental of Figure S2.png]
